# Sclerotherapy for III- and IV-degree hemorrhoids: Results of a prospective study

**DOI:** 10.3389/fsurg.2022.978574

**Published:** 2022-09-01

**Authors:** Giorgio Lisi, Paolo Gentileschi, Domenico Spoletini, Umberto Passaro, Simone Orlandi, Michela Campanelli

**Affiliations:** ^1^ Department of Surgery, Sant’Eugenio Hospital, Rome, Italy; ^2^ Department of Bariatric and Metabolic Surgery, University of Tor Vergata, San Carlo of Nancy Hospital, Rome, Italy; ^3^ Department of Gastroenterology and Digestive Endoscopy, IRCSS Sacro Cuore don Calabria, Negrar di Valpolicella, Italy; ^4^ Emergency Surgery Unit, University Hospital of Tor Vergata, Rome, Italy

**Keywords:** sclerotherapy, proctology, polidocanol 3%, bridge treatment, hemorrhoid

## Abstract

**Background:**

In the last 2 years, anorectal surgery has been strongly affected and even surgery for urgent cases cannot be scheduled; also, patients with III- and IV-degree bleeding hemorrhoids should be treated conservatively. The aim was to evaluate the effectiveness of sclerotherapy in patients who had to postpone surgery.

**Methods:**

We included all patients with III- and IV-degree bleeding hemorrhoids who underwent outpatient sclerotherapy. The visual analog scale and the hemorrhoid severity score were used at the baseline and at 4 weeks after the procedure with a telephone interview, and all patients were outpatient-evaluated 1 week, 1 month, and 1 year after the treatment. All pre- and postoperative data were recorded.

**Results:**

From October 2020 to November 2021, 19 patients with III- (12 patients; 63%) and IV-degree (7 patients; 37%) bleeding hemorrhoids were enrolled. The mean operative time was 4.5 min, and no intraoperative complications occurred. One case of tenesmus and three failures were detected. Six months after the procedure, the overall success rate was 84%, although all of the patients enrolled reported persistent bleeding at the end of the study period. Of these, 5 patients (26%) were scheduled for surgery and 11 patients (58%) refused surgery and asked to undergo a re-do sclerotherapy.

**Conclusion:**

Sclerotherapy with 3% polidocanol foam is a safe and effective procedure also in III- and IV-degree bleeding hemorrhoids. The long-term data on the length of the foam remain to be evaluated in additional studies.

## Introduction

Hemorrhoids (HDs) are one of the most frequent anorectal disorders; nevertheless, the incidence of the disease is still unclear and probably underestimated (1–5). A therapeutic strategy may vary from medical treatment to outpatient treatment and to a more invasive procedure such as hemorrhoidectomy or hemorrhoidopexy (6, 7). According to the literature, sclerotherapy (ST) and rubber band ligation (RBL) are the most common outpatient procedures for the treatment of I- and II-degree HDs among patients with failed conservative treatment; despite little is known about sclerotherapy in the III- and IV-degree hemorrhoids, few studies are published in the literature, even if still very heterogeneous, that reported the results of sclerotherapy in the treatment of I-, II- and III-degree hemorrhoids (8–10).

During the COVID-19 pandemic, 28 million procedures for benign diseases have been cancelled or rescheduled, with an estimated overall 12-week cancellation rate of 72% (11–13). All outpatient visits and operations of nononcological patients were suspended, except for highly urgent cases (14, 15). Non-COVID hospitals have been allowed outpatient proctological visits, although surgery cannot also be scheduled as a day case procedure, and even patients with several bleeding hemorrhoids should be treated conservatively. Because of this critical scenario, we recently proposed sclerotherapy for III- and IV-degree bleeding hemorrhoids as “bridge treatment” awaiting the surgical procedure proposed.

Sclerotherapy with 3% polidocanol foam induces an inflammatory reaction with sclerosis of the submucosal tissue; moreover, the obliteration of the vascular support may lead to a reduction in the hemorrhoidal volume (9, 16). Unfortunately, even if this is a reproducible and minimally invasive treatment, several life-threatening complications with liquid polidocanol have occurred (17, 18). However, from what we know, an episode of mild prostate inflammation has been detected (19).

The aim of our report was to evaluate the safety, effectiveness, and length of 3% polidocanol foam for the treatment of Sclerotherapy for III- and IV-degree hemorrhoids degree bleeding hemorrhoids in a cohort of consecutive patients during and after 1-year follow-up.

## Materials and methods

All patients above 18 and below 80 years old affected by III- and IV-degree bleeding hemorrhoids (20) with indication to surgery but postponed due to the pandemic were eligible for inclusion in this prospective study. Informed consent was submitted by all patients. Inclusion in the study was permitted only if III- and IV-degree bleeding hemorrhoidal disease could be verified by a physical examination and anoscopy. All patients were evaluated by the same surgeon (GL). Exclusion criteria are as follows: pregnancy, allergy to polidocanol, acute thrombosis, fecal incontinence, perianal fistula, anal fissure, proctitis, perianal abscess, and known hereditary thrombophilia. Pre- and postoperative data were recorded in our prospective database.

The number of bleeds per day was identified as the parameter for assessing bleeding, and it was defined as persistent in cases of more than one episode after two sclerotherapy sessions. Success was assessed as the absence of persistent bleeding. Recurrences were defined as the presence of persistent bleeding. After two ST sessions, all the patients were instructed to evaluate their postoperative pain and satisfaction with the visual analog scale (VAS) score. The hemorrhoid severity score (HSS) was used to evaluate symptoms at the baseline and 4 weeks after the treatment with a telephone interview, and all patients were examined 2 weeks, 6 months, and 1 year after the treatment (9).

All patients were submitted to clinical examination, the digital rectalexploration, and proctoscopy during the follow-up.

As “bridge treatment,” the main target of our treatment was to solve the main hemorrhoid-related symptom of bleeding, in those patients who had to postpone surgery due to the pandemic, evaluating the short-term effectiveness of sclerotherapy and after 1-year follow-up.

## Technique

**Figure 1:**
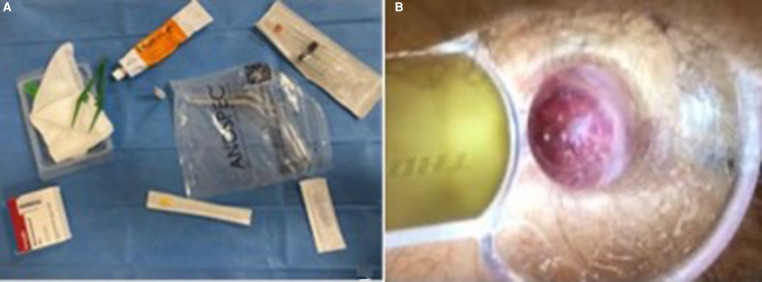
(**A**) Kit for the ST procedure: self-lighting anoscope, 20 g needle, 2 ml 3% polidocanol foam, and two 10 ml syringes. (**B**) Site of 3% polidocanol foam injection after 2 weeks.

Our procedure includes two sclerotherapy sessions 2 weeks apart to avoid discomfort due to the treatment of the three piles in a single session. We used an intravenous needle (20-G green, 0.9 mm, and 10 cc silicone syringes) of greater caliber, which made it possible to inject thicker and “creamy” foam that was obtained following the technique previously described by Tessari and subsequently by Moser and Lobascio (9, 19, 21), and the amount of foam injected for every single pile was 2 ml of 3% polidocanol (Figures 1A,B). Before each injection, the foam already obtained was re-emulsified for 45 s. All patients were treated in the Sims position (lateral decubitus position) in our outpatient clinic, injecting the foam in the two piles at 3 and 7 in the first session, then after 2 weeks at 11 o’clock, and the others if a second injection was still required. No local anesthetic nor antibiotics were used. We agree with Gallo et al. (10) on the need to use the foam in the mucosa and not in the submucosa nor the muscular layer. Furthermore, by injecting at the base of each hemorrhoidal pile above the dentate line to reduce postoperative pain, we ensured the maximum efficacy of the technique; it is necessary to inject the foam above the dentate line to avoid pain during the procedure to ensur greater effectiveness of the treatment; as emerged from recent phlebological studies, polidocanol allows tissue shrinkage and endothelium synthesis while maintaining the safety of the treatment.

## Results

A total of 19 patients with III- (*n* = 12, 63%) and IV-degree (*n* = 7, 37%) bleeding hemorrhoids with a mean age of 47 years (range 21–73 years) were consecutively enrolled and treated with 3% polidocanol foam injection. Of these, 12 (63%) patients were male and 7 were female (37%). The mean operative time was 4.5 min. No intraoperative complications or drug-related side effects were detected. All patients were discharged 10 min after the treatment. Three days after the procedure, one case of bleeding was detected. This patient was the first case with IV-degree hemorrhoids enrolled, and the bleeding was outpatient-treated with a hemostatic absorbable sponge in the anal canal removed the day after. This patient was one of the three cases where the procedure failed due to persistent bleeding after two sclerotherapy sessions (two patients with IV-degree and one patient with III-degree hemorrhoids). Finally, one case of tenesmus (III-degree) 2 days after the second ST session was detected and resolved spontaneously 2 weeks later, confirmed by the telephone interview. All patients completed the 12-month follow-up. The mean VAS after the second sclerotherapy session was 1 (range 0–1). No difference in terms of HSS comparing preoperative and postoperative symptoms at the end of the follow-up was reported (Figure 1).

Six months after the procedure, the overall success rate was 84%, although all the patients enrolled reported persistent bleeding at the end of the study period. Of these, 5 patients (26%) were scheduled for Milligan–Morgan hemorrhoidectomy because of failed treatment, but the procedure was postponed due to the pandemic, and 11 patients (58%) refused surgery and asked to undergo a re-do sclerotherapy. Finally, three patients chose to leave this study and rely on another center (Table 1). Patients with failed sclerotherapy underwent Milligan–Morgan hemorrhoidectomy as a private practice procedure.

**Table 1:** Pre- and postoperative data.

	*N* (%)
Hemorrhoid degree^a^	
III	12 (63)
IV	7 (37)
Gender	
Male	12 (63)
Female	7 (37)
Failure	3 (16)
Success rate	
After 6 months	17 (84)
After 12 months	0
End—study treatment	
Re-do—ST	11 (58)
Hemorrhoidectomy	5 (26)
Exit from the study	3 (16)

ST, sclerotherapy.

^a^ Hemorrhoid degree according to Goligher (20).

## Discussion

As highlighted by Gallo and co-authors in their recent national report, proctology was one of the most penalized surgical specialties during the outbreak, and benign anorectal disorders have been dramatically postponed. Indeed, according to a recent study including 1,050 colorectal surgeons, it emerged that proctology, surgery for benign disorders, and inpatient practice were reduced or postponed with an increased liability of malignant disease (22).

HD affects almost 5% of the western population and is a very frequent motive for attending a surgical outpatient clinic, especially in III- and IV-degree hemorrhoids (23–25). Besides causing discomfort and bleeding, these symptoms often cause restlessness, fear of cancer, and social embarrassment; Usually, this disease is not recognized as a priority today and is therefore underestimated. Due to these reasons and the need to respond to patients suffering from III- and IV-degree bleeding hemorrhoids and achieve a resolution of the bleeding, we have decided to use sclerotherapy as we cannot offer surgical therapy due to the outbreak.

The use of sclerotherapy for the treatment of hemorrhoids has risen in recent years; in fact, the strong points are cost-effectiveness, reproducibility, and the almost painless procedure, although there is a lack of homogeneous reports regarding its availability for III- and IV-degree hemorrhoids (26).

Recently, Ronconi and Colleagues (27), in their first Italian study using polidocanol foam, reported 1,427 procedures on 615 patients with a mean of 2.32 sclerotherapy sittings for each patient and a mean follow-up of 12 months. Most of the sample had II-degree HDs (317; 51.4%), and 17 (2.8%), 253 (41.1%), and 28 (4.7%) had, respectively, I-, III-, and IV-degree hemorrhoids. Furthermore, 97 patients previously underwent either excisional or nonexcisional surgery. Seventeen patients (2.7%) reported postoperative pain, which was conservatively solved.

These results were in line with another Italian retrospective report (9) concerning the use of 3% polidocanol foam in 66 patients with II- and III-degree hemorrhoids, in which the overall success rate after a single session was 78.8%; furthermore, the effectiveness reached 86% after a second sclerotherapy session. Despite these promising results, the heterogeneous sample size and absence of a control group and data on long-term effectiveness have reduced the strength of their outcomes.

Despite the small sample size, in our case series, we treated only III- and IV-degree bleeding hemorrhoids; we have chosen these patients because this was the main symptom for which they went to the hospital and asked for surgical treatment. After 6-month follow-up, compared to Gallo et al. (9), our overall success rate (84%) after two sclerotherapy sessions was lower than that reported by Moser et al. in their first experience with sclerotherapy for HD (19). However, after 1 year, all of our patients have recurrent bleeding, but we cannot compare our data at all because they treated I-degree hemorrhoids without using validated scores; moreover, according to our experience, III- and IV-degree HDs may require two sclerotherapy sessions than I-degree HDs. Moreover, the design of their report was completely different from the previous paper, which was a randomized, controlled, single-blind, and multicenter trial.

Another case series that considered sclerotherapy for I- to III-degree HDs was recently published by Salguerio et al. (28). The authors randomized 120 patients and compared rubber band ligation (RBL) and sclerotherapy, achieving an overall success rate of 88.3% with polidocanol foam and a recurrence rate of 16.1% after 1 year; despite these results were not significantly different between the groups (ST vs. RBL), the heterogeneous sample size may reduce the strength of the study.

Recently, Figueiredo et al. (29) proposed sclerotherapy with 2% polidocanol foam in 243 patients with different grades of HDs. Despite several limitations as the average follow-up, there was no information about the sample size and the number of sessions used; certainly, it cannot be considered a reliable study, although it would be interesting to compare the results with 2% and 3% polidocanol foam in terms of bleeding recurrence, effectiveness, and postprocedure complications (30).

In our case series, no life-threatening complications were reported in the perianal and prostatic areas; moreover, one case of tenesmus was spontaneously resolved. Besides, the low incidence of postoperative pain detected could be due to injection above the dentate line, which strengthens the transanal procedure compared to the endoscopic one.

Despite the small sample size, we are convinced that the strength of our study was the homogeneity of the case series and the follow-up completed by all patients. According to our experience, some considerations can be made. First, sclerotherapy with 3% polidocanol foam may be safe and effective also in III- and IV-degree HDs in terms of resolving bleeding, although we cannot be sure of the length of effectiveness; in fact, after 6 months, 84% of success rate was detected; unfortunately, all patients reported bleeding recurrence after 1 year. We believe that several studies are needed to define after how long a further session should be recommended.

Interestingly, 11 patients (58%) refused surgery and asked to undergo re-do sclerotherapy; this procedure could change the way of dealing with HDs in several patients, focusing on the main symptom reported and not on the anatomical changes due to the disease.

## Conclusion

Sclerotherapy with 3% polidocanol foam is a safe procedure in III- and IV-degree bleeding hemorrhoids also; surely, its long-term effectiveness is debated. It can be proposed in patients with or without comorbidities who could not undergo surgical treatment; however, further randomized trials are needed to confirm its use in these patients, and the long-term data on the length of the foam remain to be evaluated in additional studies.

## Data Availability

The raw data supporting the conclusions of this article will be made available by the authors without undue reservation.
